# Supplementary material for: Asymmetric Synthesis of Quaternary Hydantoins via a Palladium-Catalyzed Aza-Heck Cyclization
Source: J Am Chem Soc. 2025 Nov 14;147(49):44692–8. doi: 10.1021/jacs.5c16022 (PMC12703750; doi:10.1021/jacs.5c16022)
Supplement: Supplementary file 2 [file ja5c16022_si_002.zip › All NMR FID Files/S18/S18_CarbonNMR/2/pdata/1/email_MJE01-096-paper_2_1.pdf]

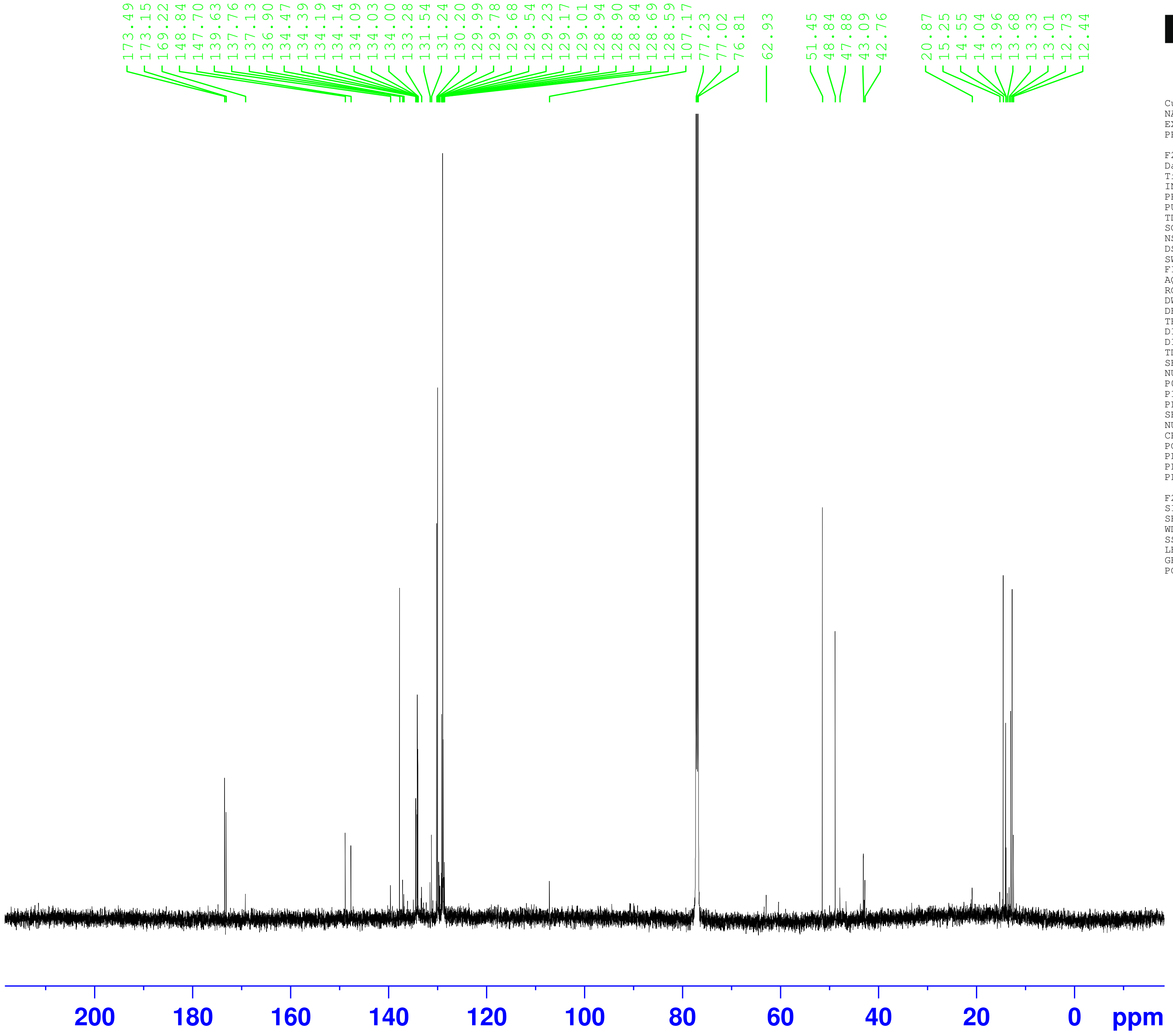


BRUKER

```
Current Data Parameters
NAME      MJE01-096-paper
EXPNO                   2
PROCNO                  1

F2 - Acquisition Parameters
Date_            20230717
Time                14.43 h
INSTRUM   Avance Neo 600 at University of Delaware
PROBHD    Z185170_0001 (
PULPROG            zgpg30
TD                  65536
SOLVENT             CDCl3
NS                   1024
DS                      4
SWH             35714.286 Hz
FIDRES           1.089913 Hz
AQ              0.9175040 sec
RG                    101
DW                 14.000 usec
DE                  18.99 usec
TE                  298.0 K
D1             2.00000000 sec
D11            0.03000000 sec
TD0                     1
SFO1          150.9178988 MHz
NUC1                  13C
P0                   3.93 usec
P1                  11.78 usec
PLW1         106.00000000 W
SFO2          600.1324005 MHz
NUC2                   1H
CPDPRG[2           waltz65
PCPD2               70.00 usec
PLW2           6.90000010 W
PLW12          0.09034800 W
PLW13          0.04544400 W

F2 - Processing parameters
SI                  32768
SF            150.9028085 MHz
WDW                    EM
SSB                     0
LB                   1.00 Hz
GB                      0
PC                   1.40
```
